# Tumor-Infiltrating Lymphocyte-Based Risk Score for Predicting Prognosis in Gastric Cancer

**DOI:** 10.3389/fonc.2020.522015

**Published:** 2020-09-30

**Authors:** Siyuan Xie, Pingfan Mo, Ning Li, Wen Cai, Jingjing Ruan, Jun Ye, Jianshan Mao

**Affiliations:** Department of Gastroenterology, The Second Affiliated Hospital, Zhejiang University School of Medicine, Hangzhou, China

**Keywords:** TIL, gastric cancer, GEO, immune risk score model, prognosis

## Abstract

Tumor-infiltrating lymphocytes (TILs) in gastric cancer are closely related to clinical prognosis; however, little is known regarding the immune microenvironment in this disease. Thus, RNA-sequencing data from gastric cancer patients were downloaded from the Gene Expression Omnibus (GEO). The proportion of immune cells was determined based on a deconvolution algorithm (CIBERSORT), and gene expression profiles were analyzed in the context of clinical outcomes to construct an immune risk score. Data were analyzed using least absolute shrinkage and selection operator (LASSO) and multivariable Cox regression, to identify prognostic markers of gastric cancer survival. The model included four immune cell types: neutrophils, plasma cells, activated CD4^+^ memory T cells, and T follicular helper cells. Patients were classified into two subgroups based on risk score, and a significant difference in overall survival (OS) was seen between the subgroups in both the training and testing cohorts, particularly in patients with tumor stages ≥T3. Multivariable analysis revealed that both T-stage and risk score were independent prognostic factors for gastric cancer survival [hazard ratio (HR) 1.505; 95% confidence interval (CI) 1.043–2.173, HR 1.686; 95% CI 1.367–2.080]. Risk scores and clinical factors were then integrated into a nomogram to build a model with both good discriminatory power and accuracy in predicting clinical outcomes. Further analysis using gene set enrichment analysis (GSEA) identified strong associations of immune risk with TGF-β and tumor metastasis-related pathways, which could inform research on the molecular mechanisms of gastric cancer. Collectively, the data presented here suggest that an immune risk model can make an important contribution to predictions prognosis in gastric cancer patients.

## Introduction

Gastric cancer is one of the most common forms of cancer worldwide, with over 1,000,000 new cases diagnosed in 2018, resulting in ∼783,000 deaths (1). In recent years, the diagnosis and treatment of early gastric cancer has progressed rapidly (2); however, the treatments for advanced stage gastric cancer remain limited. Secondary treatment with chemoradiotherapy after surgery has not provided satisfactory therapeutic results, and nor has the combination of paclitaxel with ramucirumab, an anti-VEGFR2 antibody; the overall survival (OS) time remains below 2 years (3, 4). Additional biomarkers for tumor detection and classification of gastric cancer subtypes are therefore necessary, as well as more effective treatments for advanced-stage patients.

Evidence of much greater heterogeneity in gastric cancer prognosis than previously thought is emerging from a growing number of clinical trials, even after adjusting for TNM stage. Thus, it is important to identify prognostic factors that are independent of other clinical factors, such as immune markers. As an essential component of the tumor microenvironment, immune cell infiltrates have a profound effect on tumor development and clinical outcomes. Recent studies showed that immune cell infiltration phenotypes may be associated with clinical outcomes, including tumor prognosis (5–7). Other studies revealed strong correlations between clinical outcomes and immune cells in gastric cancer, including CD8 T cells, mast cells, and tumor-associated macrophages (8, 9). Moreover, the tumor immune response has proven to be an important target of precision therapy for cancer. Immune checkpoint inhibitors have attracted significant attention in recent years, with therapies targeting immune receptors such as PD-1 and CTLA-4 being capable of limiting T cell activity by modulating various signaling pathways. As the targeting of immune checkpoint inhibitors has proven highly successful for the treatment of various cancers (10, 11), extension of this strategy to other malignancies, including gastric cancer, has become a major topic in clinical research (12).

Although previous studies have evaluated the prognostic value of single immune cell populations, comprehensive analysis of the tumor immune landscape and related molecular mechanisms has been lacking, with few studies assessing the full repertoire of immune cells present in tumor infiltrates. Such an analysis is essential both for diagnosing and understanding the progression of cancers, due to the diverse immune cell networks and highly complex interactions thereof (13). To understand the relevance of the immune response to gastric cancer, and to identify immune therapeutic targets and biomarkers, it is necessary to holistically evaluate the overall composition of tumor immune cell infiltrate. Analysis of a large number of cancer samples will increase the statistical power of any such evaluation, lending credibility to the outcomes.

Recently, a new gene expression matrix-based deconvolution algorithm known as CIBERSORT was developed, which can be used to assess the diversity characteristics of tumor-infiltrating lymphocyte (TIL) populations. CIBERSORT has proven highly effective in controlling for statistical noise and distinguishing among closely related cell types, making it a useful application for studying cell heterogeneity in multiple tissue types, including solid tumors (14). Here, we applied CIBERSORT to transcriptomic data collected from multiple tissue types, to quantify the relative proportions of 22 types of immune cells. RNA-sequencing data from gastric cancer samples were obtained from the Gene Expression Omnibus (GEO) to investigate the role of immune cells in the OS of gastric cancer patients. Least absolute shrinkage and selection operator (LASSO) and multivariate Cox regression analysis were used to establish a risk model to predict the OS of patients with gastric cancer. Gene set enrichment analysis (GSEA) revealed a strong association of immune risk with transforming growth factor (TGF)-β and tumor metastasis-related pathways, and identified immune signatures that could inform further research on the molecular mechanisms of gastric cancer (15).

## Materials and Methods

### Gastric Cancer Datasets and Processing

Gastric cancer gene expression data were obtained from the GEO database^1^. Small datasets (<50 samples) were excluded from the analysis, as were the data of patients with an OS time <1 month, or with insufficient data regarding age, gender, or TNM stage. Based on these criteria, we identified a single study (GSE84437) of patients with gastric cancer. Normalized matrix files for the dataset as well as the platform files were downloaded.

### Estimation of Immune Cell Type Fraction

To determine the proportions of the 22 infiltrating immune cells in the normalized gene expression datasets, the CIBERSORT algorithm and LM22 gene signature were used. CIBERSORT, which is a deconvolution algorithm for analyzing gene expression data, uses a series of gene expression barcodes (comprising a “signature matrix” of 547 genes) for characterizing the proportion of each immune cell type. Briefly, the gene expression datasets were uploaded to the CIBERSORT web portal^2^, and the deconvolution algorithm was run using the LM22 gene signature matrix (1,000 permutations). CIBERSORT derives a *p*-value for the deconvolution of each sample using Monte Carlo sampling, as a measure of confidence in the results; only samples with a *p-*value < 0.05 were considered for further analysis. For each sample, the sum of all estimating infiltrating immune cell fractions equaled 1.

### Immune Cell Model Construction and Verification

The CIBERSORT files were combined with relevant clinical data, and the patients were divided into training and testing cohorts according to a 1:1 ratio using a randomization method based on survival status. We included all samples with *p*-values < 0.05 in the CIBERSORT model for the training cohort analysis. LASSO regression was used to identify the most valuable prognostic immune cell subset among 22 types of immune cells, and the optimal values of the penalty parameter λ were determined by cross-validation. Associations between the proportions of immune cell types and survival were tested using multivariate Cox regression, which was also used to further filter the immune cell populations and determine the final coefficient of each cell type to construct the immune risk model. Final risk scores were calculated and “high risk” and “low risk” groups were distinguished according to the median risk score.

$$\text{Risk}\,\text{score}=\sum_{i=1}^{n}{\text{Coefi}}^{*}\,\text{Fractioni}$$

The associations of infiltrating immune cell subsets with OS were analyzed using Kaplan–Meier curves, with receiver operating characteristic (ROC) curve analysis used to verify the sensitivity and specificity of the model for the training cohort. The immune risk model was then applied to the testing cohort and Kaplan–Meier and ROC curve analyses were used to verify the reliability of the model.

Immune cell populations were further classified based on tumor stage, with matrices isolated from T1 and T2 tumors divided into one cohort and T3 and T4 tumors into another. The risk model was then applied to each cohort and “high risk” and “low risk” groups were distinguished. Kaplan–Meier curves were plotted for each cohort to illustrate the gastric cancer stage most suitable for application of the model.

### Independent Prognostic Factors and Clinical Prognosis Model

Univariate and multivariate Cox regression analyses were used to evaluate the correlations between OS and clinical factors, and to identify factors independently predicting disease outcomes.

The independent prognostic factors were then used to construct a new clinical model. The data were visualized using a nomogram to assess the relationship between the variables in the clinical model and prognosis. Harrell’s concordance index (C-index) was calculated and the prognostic models were calibrated using the R survival package.

### GSEA

Gene set enrichment analysis was used to identify differences in gene enrichment between the low- and high-risk cohorts based on Gene Ontology (GO) and Kyoto Encyclopedia of Genes and Genomes (KEGG) pathway analysis, and on immunologic signatures.

### Statistical Analysis

Statistical analysis was conducted using R software (version 3.6.1; R Foundation for Statistical Computing, Vienna, Austria). All statistical tests were two-sided, with *p*-values < 0.05 considered statistically significant.

## Results

### Flowchart

**FIGURE 1:**
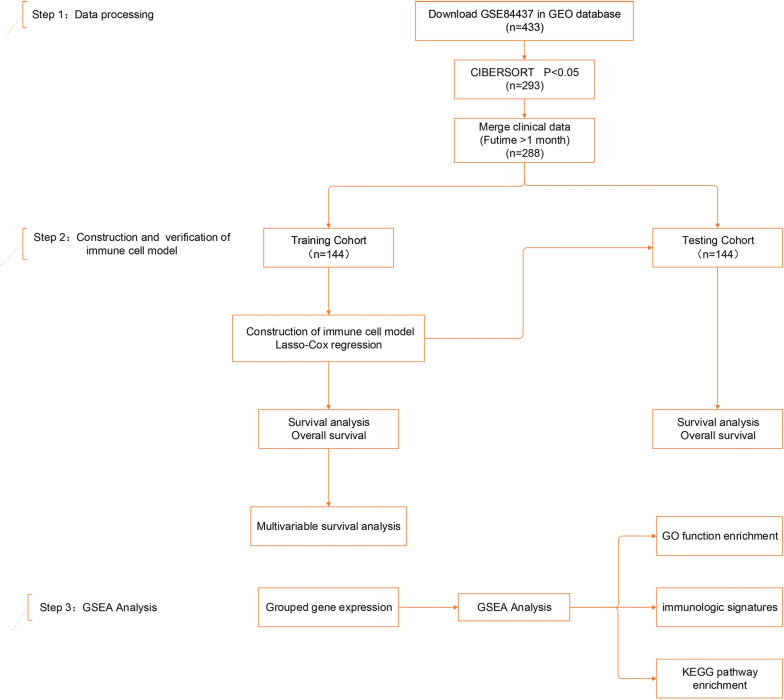
Study design and patient demographics. Step 1: The dataset of study GSE84437, containing 433 gastric cancer tissue samples, was mined using CIBERSORT. Data were filtered based on *p*-values and clinical outcomes, yielding a final cohort of 288 patients. Patients were divided into training and testing cohorts according to 1:1 ratio using a randomization method. Step 2: Four infiltrating immune cell types in the training cohort were screened by least absolute shrinkage and selection operator (LASSO) and multivariate Cox regression, and validated in both the training and testing cohorts. Step 3: Gene set enrichment analysis (GSEA) analysis was used to identify subgroup differences in gene enrichment.

A total of 288 clinically annotated gastric cancer samples were identified that met the screening criteria described above. Supplementary Table S1 provides a summary of the immune cell compositions within and across gastric cancer subgroups. A schematic overview is shown in Figure 1.

### Establishment of the Immune Risk Model

**FIGURE 2:**
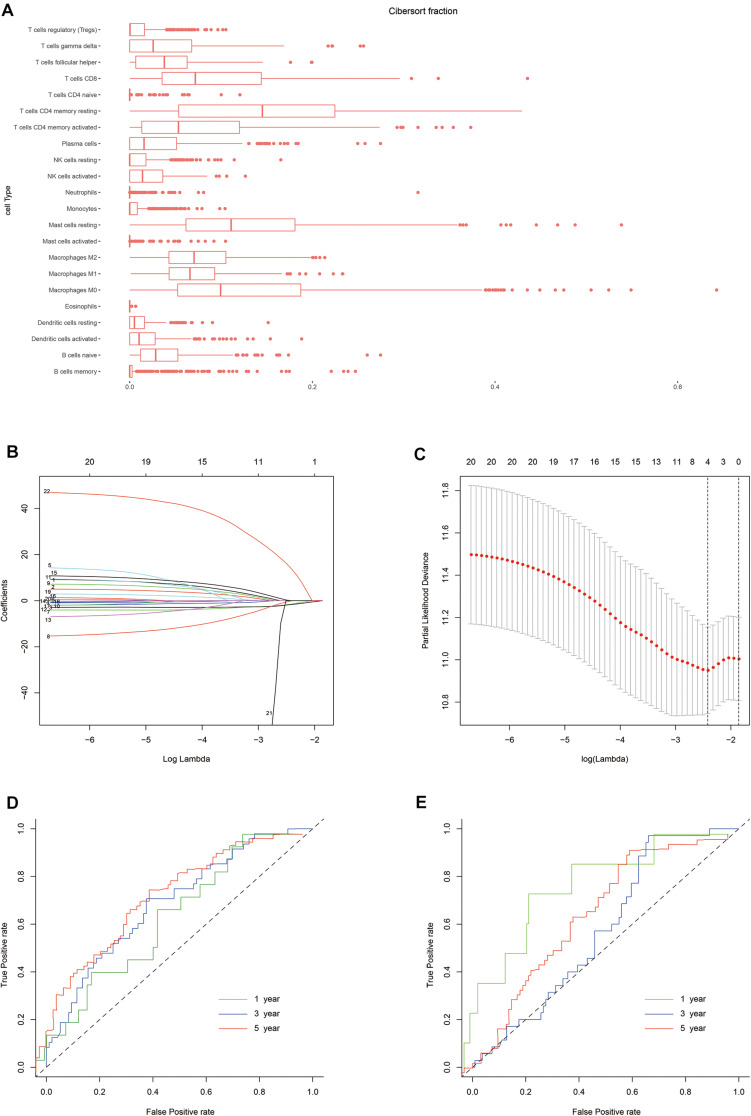
Construction of the risk model. **(A)** Box plot showing the proportions of 21 immune cell types across 288 samples filtered by CIBERSORT. **(B)** LASSO regression coefficients of the 21 immune cell types. The dotted line indicates the value chosen by tenfold cross-validation. **(C)** Tenfold cross-validation informing the parameter selection in the LASSO model. **(D,E)** Risk score determined by time-dependent receiver operating characteristic (ROC) curves for the training and testing cohorts. The area under the ROC curve was 0.600, 0.691, and 0.716 for the risk scores for 1-, 3-, and 5-year overall survival (OS) in the training cohort, versus 0.800, 0.580, and 0.632 in the testing cohort, respectively.

The normalized gene expression profiles of human gastric cancer cells were analyzed using CIBERSORT. The proportions of the 22 immune cell types are shown in Figure 2. To evaluate the association between immune characteristics and prognostic outcomes, four features were extracted from among the 22 different immune cell types in the training cohort using LASSO regression (λ = −4). The partial likelihood deviance for this penalty parameter was 10.949 (minimum deviance). Next, stepwise regression was used to further filter the different immune cell types and identify the optimal coefficient for each population. Finally, four cell types were selected to construct the immune risk model. The formula of the model was as follows: risk score = 35.127 ^∗^ neutrophils – 5.798 ^∗^ plasma cells – 4.155 ^∗^ activated CD4^+^ memory T cells - 6.239 ^∗^ T follicular helper (Tfh) cells. The training and testing cohorts were both divided equally into high and low risk groups using the above formula, according to the median risk score in the training cohort.

### Validation of the Risk Model for Predicting Survival

**FIGURE 3:**
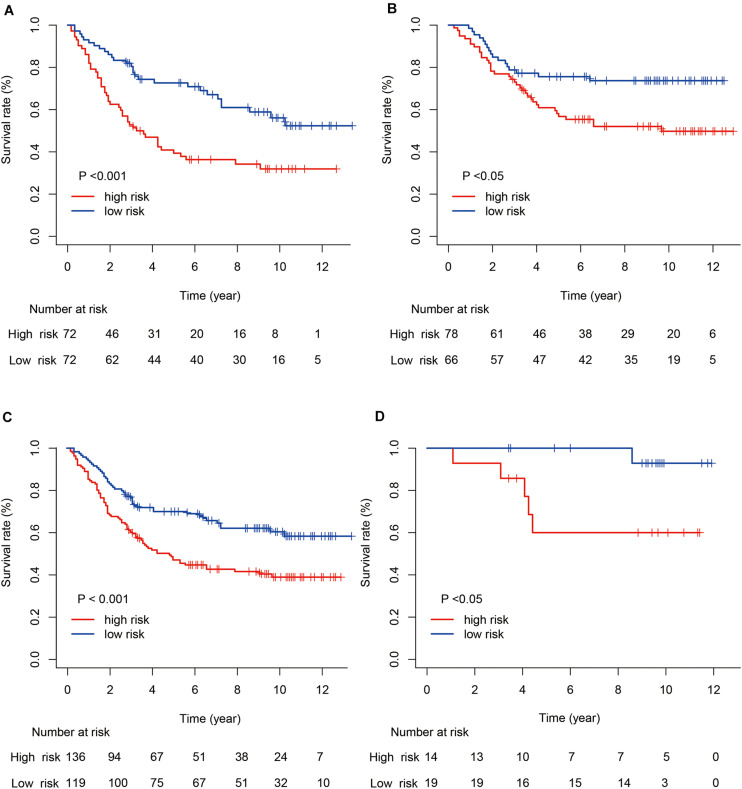
Kaplan–Meier survival curves for gastric cancer risk score and tumor (T) stage. **(A,B)** Kaplan–Meier survival curves for the training and testing cohorts. **(C,D)** Kaplan–Meier survival curves for stages T1–T2 and T3–T4.

Next, we evaluated the prognostic value of the immune risk model with respect to OS. Cases in the high-risk group had a significantly worse OS relative to the low risk group, both in the training and testing cohorts (*p* < 0.001 and *p* < 0.05, respectively; Figure 3). The 1-, 3-, and 5-year OS rates were 0.896, 0.639, and 0.486 in the training cohort and 0.965, 0.854, and 0.750 in the testing cohort, respectively. The accuracy of the model was verified using time-dependent ROC curves, which confirmed the reliability of the prognoses in both cohorts. The area under the ROC curve for the risk score was 0.600, 0.691, and 0.716 for 1-, 3- and 5-year OS in the training cohort, versus 0.800, 0.580 and 0.632 in the testing cohort, respectively. Next, the training and testing cohorts were combined to derive a new cohort and then re-divided into two subgroups according to tumor (T) stage. The degree of separation between the two groups, according to Kaplan–Meier survival curves, showed a significant difference between the T1–T2 (*p* = 0.02) and T3–T4 patients (*p* < 0.001).

### Characteristics of the Immune Risk Score Model

**FIGURE 4:**
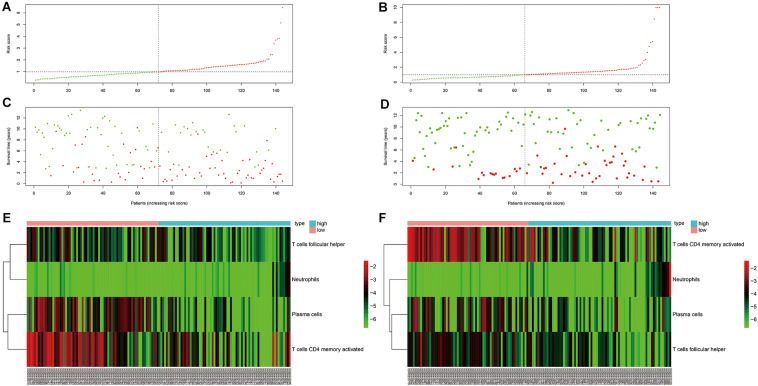
Risk curve and heatmap grouped according to risk score in the training and testing cohorts. **(A,C,E)** Risk curve and selected immunocyte populations in the training cohort. **(B,D,F)** Risk curve and selected immunocyte populations in the testing cohort.

As can be seen in Figure 4, OS was obviously different between subgroups for both the training and testing cohorts. Green and red areas correspond to low and high risk scores, respectively. The heatmaps demonstrated clear segregation of the four selected immune cells into two subgroups. The proportions of activated CD4^+^ memory T cells, plasma cells, and Tfh cells in the high risk score group were significantly higher than in the low risk score group, with the opposite pattern seen for neutrophils.

### Independent Prognostic Factors of Gastric Cancer

Clinical parameters are crucial for accurate patient prognosis. In this study, univariate Cox regression analysis was first conducted to evaluate the correlations between survival prognosis and clinical factors. T stage and risk score were the first factors tested, and both proved to be negative predictors of survival [hazard ratio (HR) 1.505, 95% confidence interval (CI) 1.043–2.173; HR 1.686, 95% CI 1.367–2.080]. These factors were also identified as independent prognostic factors by multivariate Cox regression (HR 1.468, 95% CI 1.010–2.135; HR 1.658, 95% CI 1.331–2.065). The HRs for T stage and risk score were both >1, showing that these factors were strongly associated with poorer survival outcomes.

### Nomogram of Independent Factors

**FIGURE 5:**
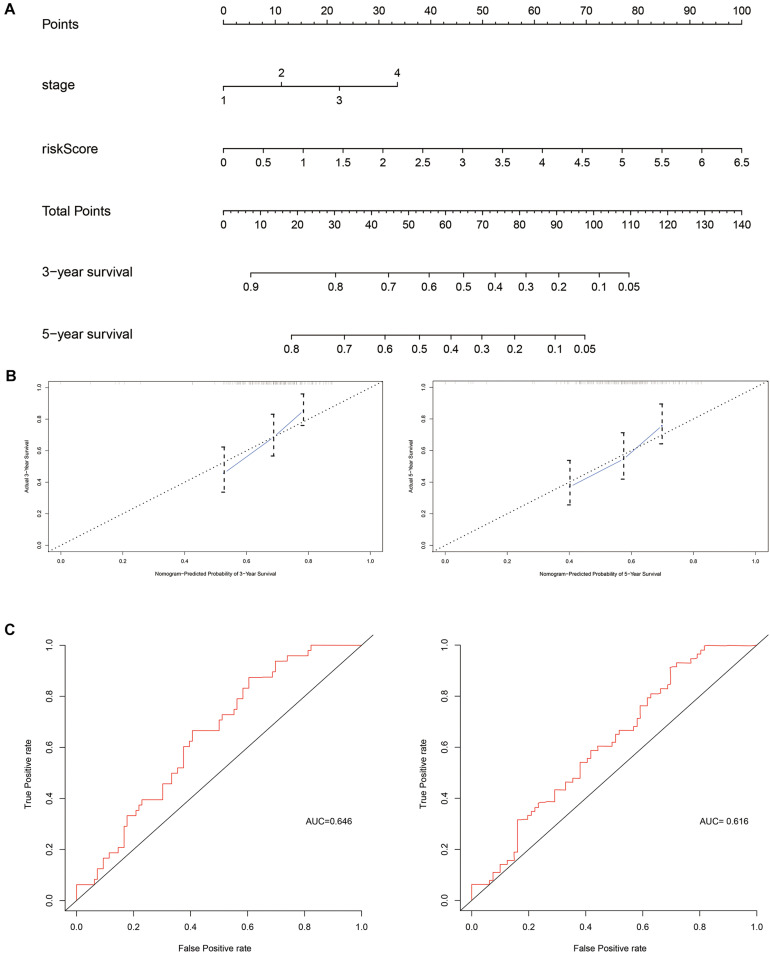
Evaluation of independent prognostic factors in the training cohort. **(A)** Nomogram of independent prognostic factors including risk score and T stage. The values attributed to each individual patient are located on the variable axis; the upward line shows the points received for each variable. The sum of the scores is shown on the total points axis; the line drawn downward to the survival axis shows the likelihood of 3- or 5-year survival. **(B)** The calibration curve for predicting 3- and 5-year OS in the training cohort. **(C)** The time-dependent ROC curves for 3- and 5-year OS in the training cohort.

Next, independent prognostic factors were used to construct a new clinical model. A nomogram was built that integrated the T stage and risk score to predict 3- and 5-year OS in the training cohort (Figure 5). The C-index of the nomogram was 0.662. The calibration curves showed satisfactory predictive power with respect to 3- and 5-year OS, as determined by comparing the actual observations against the predictions.

### Differences in Gene Enrichment Between Immune Risk Subgroups

**FIGURE 6:**
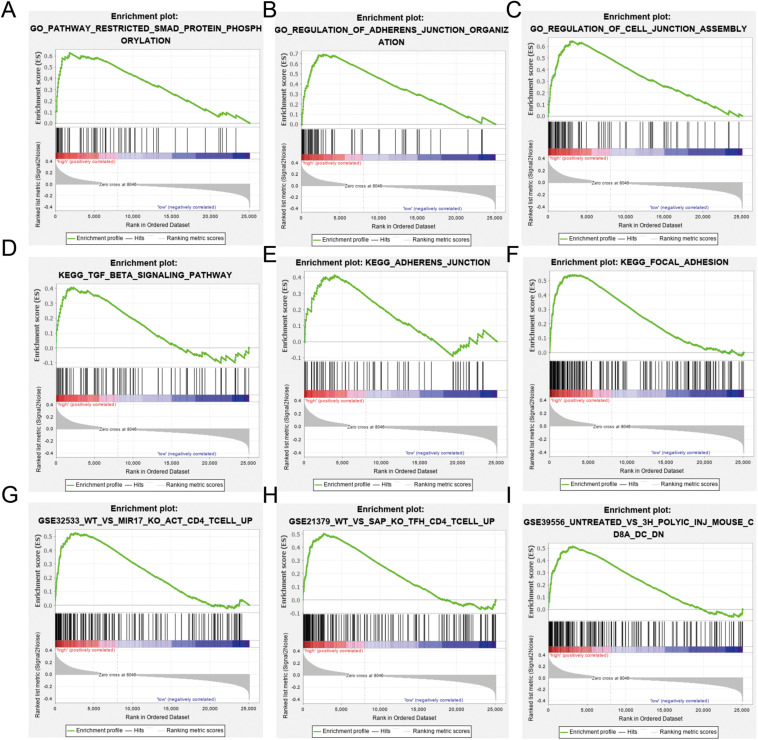
Gene Ontology (GO), Kyoto Encyclopedia of Genes and Genomes (KEGG), and immunologic signature analysis using gene set enrichment analysis (GSEA) according to risk subgroup in the merged dataset. NES, normalized enrichment score; NOM-p, nominal *p*-value; FDR-q, false discovery rate. **(A–C)** SMAD protein phosphorylation, adherens junction and cell junction enriched in the GO analysis. **(D–F)** Transforming growth factor (TGF)-β signaling, adherens junction, and focal adhesion pathway enriched in the KEGG analysis. **(G–I)** Immunologic signature analysis.

Gene set enrichment analysis was used to identify differences in gene enrichment between low- and high-risk cohorts based on GO, and KEGG pathway analysis, and immunologic signatures (Figure 6). Several biological pathways associated with malignant tumor phenotypes were significantly enriched in the high risk group, including focal adhesion [normalized enrichment score (NES) = 1.91, *p* < 0.001, false discovery rate (FDR) = 0.016], TGF-β signaling (NES = 1.48, *p* = 0.038, FDR = 0.224), and leukocyte transendothelial migration (NES = 1.39, *p* = 0.067, FDR = 0.234) in KEGG pathways. GO analysis revealed significant up-regulation of adherens junction organization (NES = 2.11, *p* < 0.001, FDR = 0.005) and positive regulation of SMAD protein phosphorylation (NES = 2.16, *p* < 0.001, FDR = 0.005) in activated CD4^+^ memory T cells compared to study GSE32533 (NES = 1.93, *p* < 0.001, FDR = 0.046), a study validating miR-17 from the miR-17-92 cluster regulating activation-induced cell death in T cells and modulating inducible regulatory T cell differentiation. Genes upregulated in CD4^+^ Tfh cells formed a unique immunologic signature compared to study GSE21379 (NES = 1.85, *p* < 0.001, FDR = 0.064), a study revealing a prominent role for SLAM receptor ligation in IL-4 production by germinal center CD4 T cells but not in Tfh and GC Tfh differentiation. For the low risk group, natural killer (NK) cell-mediated cytotoxicity (NES = −1.75, *p* = 0.010, FDR = 0.069) and T cell receptor signaling (NES = −1.67, *p* = 0.026, FDR = 0.093) were shown to be upregulated based by KEGG analysis, while positive regulation of NK cell-mediated immunity (NES = −0.80, *p* < 0.001, FDR < 0.001) and myosin heavy chain (MHC) protein binding (NES = −0.74, *p* = 0.004, FDR = 0.072) were significant based on GO analysis.

## Discussion

Our risk model, as a novel prognostic tool designed to improve the accuracy of survival predictions for patients with gastric cancer, was established and validated in this retrospective study. LASSO and multivariate COX regression analyses of four immune cell types, including activated CD4^+^ memory T cells, plasma cells, neutrophils, and Tfh cells, were performed, with significant correlations with OS seen for all cell types except Tfh cells (*p* < 0.05). Due to the strong correlation between infiltrating immune cell types seen in this study, LASSO regression was essential for reducing collinearity through selection of the optimal penalty parameter λ, after which stepwise regression with the Akaike information criterion (AIC) was applied to filter immune cells and select the optimal coefficient for each cell population.

Previous studies revealed that memory T cells could provide protection against both *Helicobacter pylori* infection and gastric cancers, and were shown to be associated with lymph node metastases in gastric cancer (16, 17). As CD4^+^ memory T cells are the most abundant immune cells in tumor tissues, more research into their role in tumor metastasis and disease progression is necessary; these cells represent a potential target for immune therapy. In this study, infiltration of plasma cells was also shown to prolong survival in gastric cancer as a component of the humoral immune response, consistent with previous studies (18, 19); however, the precise role of these cells remains poorly understood. Neutrophils were assigned the highest coefficient in our risk assessment formula, possibly due to their ability to regulate many of the malignancy-associated behaviors of cancer cells, such as migration and invasion (20, 21). The difference in infiltration ratio among these four immune cell types may be correlated with leukocyte migration-related pathways, which were enriched in our high risk subgroup; a high risk score was indicative of infiltrates with a high proportion of neutrophils and low proportions of plasma cells, activated CD4^+^ memory T cells, and Tfh cells. However, how this influences clinical outcomes remains unclear, although methods such as GSEA do suggest a variety of possible molecular mechanisms (see below).

Stratified analyses were performed by T stage, and Kaplan–Meier curves showed clear segregation between subgroups (T1–T2 vs. T3–T4), indicating that our model may be more useful for more advanced cases of gastric cancer. Furthermore, the contribution of the risk score to the prognosis of each subgroup was significant: the risk score was an independent prognostic factor and could therefore be used to supplement the established prognostic factor of T stage. The reason why the model appears more suitable for advanced-stage patients may be the higher proportion of T3–T4 stage samples in the training cohort used to construct the model.

The molecular mechanisms and biological processes underlying the impact of the immune cell infiltration ratio on survival were also investigated. GO, and KEGG GSEA analyses were used due to the limited number of differentially expressed genes between the two subgroups. It is worth noting that both the GO and KEGG GSEA results showed significant enrichment in the regulation of adherens junction organization and focal adhesion in the high risk subgroup, suggesting that a change in the proportions of infiltrating immune cell types may influence the risk of tumor metastasis and, by extension, the prognosis. TGF-β signaling and positive regulation of SMAD protein phosphorylation were also enriched in this subgroup based on both GO and KEGG analyses. Thus, we suspect that the TGF-β signaling pathway may interact with SMAD phosphorylation to affect tumor metastasis. This is strongly supported by a recent study showing that tumor-derived TGF-β could suppress the anti-tumor function of CD4^+^ T cells through SMAD protein phosphorylation in the tumor effusion fluids of metastatic patients (22). Also, a known determinant of PD-1/PD-L1 immunotherapy outcomes is TGF-β pathway regulation, which can restrain anti-tumor immunity by restricting T cell infiltration (23).

NK cell-mediated immunity was associated with our low risk group, with neutrophils potentially influencing the clinical outcome of gastric cancer via regulation of this immune response (24); this is consistent with neutrophils having the highest coefficient in our risk assessment model. Indeed, enriched neutrophils have been shown to induce NK cell activation via receptor-ligand interactions (25) and production of interleukin (IL)-18 (26).

The immunologic signature analysis described herein was accomplished via GSEA. Enrichment of miR-17-regulated genes in activated CD4^+^ T cells was shown to facilitate CD4^+^ T cell expansion, and to modulate cell death and the differentiation of regulatory T cells (27). As the activated CD4^+^ memory T cell population in this risk model was shown to be a protective factor (HR 0.0157, 95% CI 0.0007–0.3394), this phenomenon could be interpreted as a byproduct of miR-17 activity. The miR-17 cluster is essential for a T cell-mediated anti-tumor response *in vivo*, through strict enforcement of Th1 lineage–specific functions (28, 29). Similarly, SH2D1A-regulated genes in CD4^+^ Tfh cells, as well as genes up-regulated in CD4^+^ memory T cells (27), were also enriched. Through adoptive transfer of antigen-specific subpopulations of CD4^+^memory T cells, distinct CD4^+^ memory T cell populations committed to Tfh lineages could be identified (28). As Tfh cells are essential for the development of germinal center, this population could provide the signals required by B cells to facilitate maturation (30). The above findings may explain why the activated CD4^+^ memory T cells and plasma cells were identified as protective by our LASSO-COX regression analyses (HR 0.016, 95% CI 0.0007–0.3394; HR 0.003, 95% CI 0.001–0.642). Beyond these observations, many similar immunologic signatures remained to be investigated.

Despite the findings detailed above, there were also some limitations to our analysis. First, the accuracy of the model remains suboptimal, as the area under the ROC curves for both the training and testing cohorts was <0.8. Second, the GSEA of the molecular mechanisms underlying the relationship between risk score and clinical outcomes was not robust. Moreover, even though the results suggested that tumor metastasis may be well explained by our model, it was difficult to validate this due to the absence of N and M stage data. Further research may provide greater insight into the mechanisms driving gastric cancer. Finally, the conclusions of this study were based on bioinformatic analyses only. Whether the model could be used to predict patient survival based on tumor biopsies remains unclear. Further validation of our findings is necessary using experimental data.

In summary, we analyzed 22 distinct immune cells present in the tumor infiltrates of gastric cancer and established an immune risk model which is not only a vital supplement to the prognostic prediction of gastric cancer but also providing a new direction for the subsequent targeted therapy in patients with high immunological risk. On this basis, a new clinical prognosis prediction model was constructed combining the immune risk model with clinical data. Thus, the new clinical model might have crucial implication in predicting prognosis of postoperative gastric cancer.

## Data Availability Statement

Publicly available datasets were analyzed in this study, these can be found in the NCBI Gene Expression Omnibus (GSE84437).

## Author Contributions

JM and JY designed and conceived the study. SX and PM performed the data analyses and wrote the manuscript for the study. NL, WC, and JR downloaded the data from GEO database. All authors contributed to the reviewing of the manuscript, and approved the final manuscript for submission.

## Conflict of Interest

The authors declare that the research was conducted in the absence of any commercial or financial relationships that could be construed as a potential conflict of interest.

## Footnotes

1 https://www.ncbi.nlm.nih.gov/geo/

2 http://cibersort.stanford.edu/

## References

[1] Bray F Ferlay J Soerjomataram I Siegel RL Torre LA Jemal A. Global cancer statistics 2018: GLOBOCAN estimates of incidence and mortality worldwide for 36 cancers in 185 countries. *CA Cancer J Clin.* (2018) 68:394–424. 10.3322/caac.21492 30207593

[2] Brierley R. ESD for early gastric cancer. *Lancet Gastroenterol.* (2017) 2:474–474.

[3] Cats A Jansen EPM van Grieken NCT Sikorska K Lind P Nordsmark M Chemotherapy versus chemoradiotherapy after surgery and preoperative chemotherapy for resectable gastric cancer (CRITICS): an international, open-label, randomised phase 3 trial. *Lancet Oncol.* (2018) 19:616–28. 29650363 10.1016/S1470-2045(18)30132-3

[4] Wilke H Muro K Van Cutsem E Oh SC Bodoky G Shimada Y Ramucirumab plus paclitaxel versus placebo plus paclitaxel in patients with previously treated advanced gastric or gastro-oesophageal junction adenocarcinoma (RAINBOW): a double-blind, randomised phase 3 trial. *Lancet Oncol.* (2014) 15:1224–35. 10.1016/s1470-2045(14)70420-6 25240821

[5] Ali HR Chlon L Pharoah PDP Markowetz F Caldas C. Patterns of immune infiltration in breast cancer and their clinical implications: a gene-expression-based retrospective study. *PLoS Med.* (2016) 13:e1002194. 10.1371/journal.pmed.1002194 27959923 PMC5154505

[6] Rohr-Udilova N Klinglmuller F Schulte-Hermann R Stift J Herac M Salzmann M Deviations of the immune cell landscape between healthy liver and hepatocellular carcinoma. *Sci. Rep.* (2018) 8:6220. 10.1038/s41598-018-24437-5 PMC5906687 29670256

[7] Xiong YF Wang K Zhou H Peng LL You WX Fu ZX. Profiles of immune infiltration in colorectal cancer and their clinical significant: a gene expression-based study. *Cancer Med.* (2018) 7:4496–508. 10.1002/cam4.1745 30117315 PMC6144159

[8] Sakamoto S Kagawa S Kuwada K Ito A Kagawa T Kikuchi S Tumor associated macrophages promote malignant phenotypes of disseminated human gastric cancer cells in intraperitoneal cancer immune microenvironment. *Cancer Res.* (2017) 77:5011.

[9] Thompson ED Zahurak M Murphy A Cornish T Cuka N Abdelfatah E Patterns of PD-L1 expression and CD8 T cell infiltration in gastric adenocarcinomas and associated immune stroma. *Gut.* (2017) 66:794–801. 10.1136/gutjnl-2015-310839 26801886 PMC4958028

[10] Dyck L Mills KHG. Immune checkpoints and their inhibition in cancer and infectious diseases. *Eur J Immunol.* (2017) 47:765–79. 10.1002/eji.201646875 28393361

[11] Kudo M. Immune checkpoint inhibition in hepatocellular carcinoma: basics and ongoing clinical trials. *Oncology.* (2017) 92:50–62. 10.1159/000451016 28147363

[12] Smyth E Thuss-Patience PC. Immune checkpoint inhibition in gastro-oesophageal cancer. *Oncol Res Treat.* (2018) 41:272–80. 10.1159/000489099 29705787

[13] Udall M Rizzo M Kenny J Doherty J Dahm S Robbins P PD-L1 diagnostic tests: a systematic literature review of scoring algorithms and test-validation metrics. *Diagn Pathol.* (2018) 13:12. 10.1186/s13000-018-0689-9 PMC5807740 29426340

[14] Newman AM Liu CL Green MR Gentles AJ Feng WG Xu Y Robust enumeration of cell subsets from tissue expression profiles. *Nat Methods.* (2015) 12:453–7. 10.1038/nmeth.3337 25822800 PMC4739640

[15] Subramanian A Tamayo P Mootha VK Mukherjee S Ebert BL Gillette MA Gene set enrichment analysis: a knowledge-based approach for interpreting genome-wide expression profiles. *Proc Natl Acad Sci USA.* (2005) 102:15545–50. 10.1073/pnas.0506580102 16199517 PMC1239896

[16] Mueller SN Mackay LK. Tissue-resident memory T cells: local specialists in immune defence. *Nat Rev Immunol.* (2016) 16:79–89. 10.1038/nri.2015.3 26688350

[17] Liu W Zeng ZQ Luo SH Hu CP Xu NY Huang A Gastric subserous vaccination with *Helicobacter* pylori vaccine: an attempt to establish tissue-resident CD4+memory T cells and induce prolonged protection. *Front Immunol.* (2019) 10:1115. 10.3389/fimmu.2019.01115 31156652 PMC6533896

[18] Zhang R Li F Li H Yu J Ren X. The clinical significance of memory T cells and its subsets in gastric cancer. *Clin Transl Oncol.* (2014) 16:257–65. 10.1007/s12094-013-1066-5 23793812

[19] Fristedt R Borg D Hedner C Berntsson J Nodin B Eberhard J Prognostic impact of tumour-associated B cells and plasma cells in oesophageal and gastric adenocarcinoma. *J Gastrointest Oncol.* (2016) 7:848–59. 10.21037/jgo.2016.11.07 28078109 PMC5177573

[20] Zhang W Gu J Chen J Zhang P Ji R Qian H Interaction with neutrophils promotes gastric cancer cell migration and invasion by inducing epithelial-mesenchymal transition. *Oncol Rep.* (2017) 38:2959–66. 10.3892/or.2017.5942 28901479

[21] Li S Cong X Gao H Lan X Li Z Wang W Tumor-associated neutrophils induce EMT by IL-17a to promote migration and invasion in gastric cancer cells. *J Exp Clin Cancer Res.* (2019) 38:6. 10.1186/s13046-018-1003-0 PMC6323742 30616627

[22] Dimeloe S Gubser P Loeliger PJ Frick C Develioglu L Fischer P Tumor-derived TGF-β inhibits mitochondrial respiration to suppress IFN-γ production by human CD4+ T cells. *Sci Signal.* (2019) 12:eaav3334. 10.1126/scisignal.aav3334 31530731

[23] Mariathasan S Turley SJ Nickles D Castiglioni A Yuen K Wang Y TGF beta attenuates tumour response to PD-L1 blockade by contributing to exclusion of T cells. *Nature.* (2018) 554:544–8. 29443960 10.1038/nature25501 PMC6028240

[24] Herbst RS Soria JC Kowanetz M Fine GD Hamid O Gordon MS Predictive correlates of response to the anti-PD-L1 antibody MPDL3280A in cancer patients. *Nature.* (2014) 515:563–7. 25428504 10.1038/nature14011 PMC4836193

[25] Amano K Hirayama M Azuma E Iwamoto S Keida Y Komada Y. Neutrophils induced licensing of natural killer cells. *Mediat Inflamm.* (2015) 2015:747680. 10.1155/2015/747680 PMC4419236 25977601

[26] Sporri R Joller N Hilbi H Oxenius A. A novel role for neutrophils as critical activators of NK cells. *J Immunol.* (2008) 181:7121–30. 10.4049/jimmunol.181.10.7121 18981133

[27] Jiang S Li C Olive V Lykken E Feng F Sevilla J Molecular dissection of the miR-17-92 cluster’s critical dual roles in promoting Th1 responses and preventing inducible Treg differentiation. *Blood.* (2011) 118:5487–97. 10.1182/blood-2011-05-355644 21972292 PMC3217351

[28] Tumeh PC Harview CL Yearley JH Shintaku IP Taylor EJM Robert L PD-1 blockade induces responses by inhibiting adaptive immune resistance. *Nature.* (2014) 515:568–71. 25428505 10.1038/nature13954 PMC4246418

[29] Hamid O Robert C Daud A Hodi FS Hwu WJ Kefford R Genetic basis for clinical response to CTLA-4 blockade in Melanoma (vol 371, pg 2189, 2014). *N Engl J Med.* (2018) 379:2185–2185. 10.1056/nejmx180040 25409260 PMC4315319

[30] Haxhinasto S Mathis D Benoist C. The AKT-mTOR axis regulates de novo differentiation of CD4(+)Foxp3(+) cells. *J Exp Med.* (2008) 205:565–74. 10.1084/jem.20071477 18283119 PMC2275380

