# Supplementary material for: Tumor-Infiltrating Lymphocyte-Based Risk Score for Predicting Prognosis in Gastric Cancer
Source: Front Oncol. 2020 Sep 30;10:522015. doi: 10.3389/fonc.2020.522015 (PMC7561394; doi:10.3389/fonc.2020.522015)
Supplement: Supplementary file 5 [file Table_5.DOCX]

**#box plot**

setwd("C:\\Users\\BOSS\\Desktop\\new-gene\\hist")

rt=read.table("cibersort.txt",sep="\t",header=T,check.names=F)

library(reshape2)

rt1 <- melt(rt,id.vars ='ID')

rt2 <- rt1[,-2]

pdf()

library(ggplot2)

pdf("boxplot.pdf",height=8,width=15)

ggplot(rt2,aes(x=ID,y=value,colour='Orange'))+geom_boxplot()+labs(title='Cibersort fraction',x='cell Type',y='')+

coord_flip()+theme_bw()+ theme(plot.title = element_text(hjust = 0.5))+ theme(panel.grid.major = element_blank(),panel.grid.minor=element_blank(),panel.border = element_blank(),legend.position="none")

dev.off()

**#Group to training cohort and testing cohort**

install.packages("caret")

setwd("C:\\Users\\lexb4\\Desktop\\coxTest\\04.trainTest")

rt=read.table("expTime.txt",sep="\t",header=T,check.names=F)

library(caret)

inTrain<-createDataPartition(y=rt[,3],p=0.5,list=F)

train<-rt[inTrain,]

test<-rt[-inTrain,]

write.table(train,file="train.txt",sep="\t",quote=F,row.names=F)

write.table(test,file="test.txt",sep="\t",quote=F,row.names=F)

**#LASSO regression**

setwd("C:\\Users\\BOSS\\Desktop\\last\\loop")

rt=read.table("04.train.txt",header=T,sep="\t",row.names=1,check.names=F)

x=as.matrix(rt[,c(3:ncol(rt))])

y=data.matrix(Surv(rt$futime,rt$fustat))

fit <- glmnet(x, y, family = "cox", maxit = 1000)

pdf("lambda.pdf")

plot(fit, xvar = "lambda", label = TRUE)

dev.off()

cvfit <- cv.glmnet(x, y, family="cox", maxit = 1000)

pdf("cvfit.pdf")

plot(cvfit)

abline(v=log(c(cvfit$lambda.min,cvfit$lambda.1se)),lty="dashed")

dev.off()

coef <- coef(fit, s = cvfit$lambda.min)

index <- which(coef != 0)

actCoef <- coef[index]

lassoGene=row.names(coef)[index]

lassoGene=c("futime","fustat",lassoGene)

lassoSigExp=rt[,lassoGene]

lassoSigExp=cbind(id=row.names(lassoSigExp),lassoSigExp)

write.table(lassoSigExp,file="lassoSigExp.txt",sep="\t",row.names=F,quote=F)

**#MultiCox Regression**

setwd("C:\\Users\\BOSS\\Desktop\\last\\loop")

install.packages('survival')

install.packages("survminer",dependencies = T)

library(survival) # risk model construction and verification

library(survminer)

rt=read.table("lassoSigExp.txt",header=T,sep="\t",check.names=F,row.names=1)

multiCox=coxph(Surv(futime, fustat) ~ ., data = rt)

multiCox=step(multiCox,direction = "both")

multiCoxSum=summary(multiCox)

outTab=data.frame()

outTab=cbind(

coef=multiCoxSum$coefficients[,"coef"],

HR=multiCoxSum$conf.int[,"exp(coef)"],

HR.95L=multiCoxSum$conf.int[,"lower .95"],

HR.95H=multiCoxSum$conf.int[,"upper .95"],

pvalue=multiCoxSum$coefficients[,"Pr(>|z|)"])

outTab=cbind(id=row.names(outTab),outTab)

write.table(outTab,file="multiCox.xls",sep="\t",row.names=F,quote=F)

pdf(file="forest.pdf",

width = 8,

height = 5,

)

ggforest(multiCox,

main = "Hazard ratio",

cpositions = c(0.02,0.22, 0.4),

fontsize = 0.7,

refLabel = "reference",

noDigits = 2)

dev.off()

riskScore=predict(multiCox,type="risk",newdata=rt)

coxGene=rownames(multiCoxSum$coefficients)

coxGene=gsub("`","",coxGene)

outCol=c("futime","fustat",coxGene)

medianTrainRisk=median(riskScore)

risk=as.vector(ifelse(riskScore>medianTrainRisk,"high","low"))

write.table(cbind(id=rownames(cbind(rt[,outCol],riskScore,risk)),cbind(rt[,outCol],riskScore,risk)),

file="riskTrain.txt",

sep="\t",

quote=F,

row.names=F)

rtTest=read.table("04.test.txt",header=T,sep="\t",check.names=F,row.names=1)

riskScoreTest=predict(multiCox,type="risk",newdata=rtTest)

riskTest=as.vector(ifelse(riskScoreTest>medianTrainRisk,"high","low"))

write.table(cbind(id=rownames(cbind(rtTest[,outCol],riskScoreTest,riskTest)),cbind(rtTest[,outCol],riskScore=riskScoreTest,risk=riskTest)),

file="riskTest.txt",

sep="\t",

quote=F,

row.names=F)

**# Validation of the risk model for predicting survival**

library(survival)

rt=read.table("riskTrain.txt",header=T,sep="\t")

diff=survdiff(Surv(futime, fustat) ~risk,data = rt)

pValue=1-pchisq(diff$chisq,df=1)

pValue=signif(pValue,4)

pValue=format(pValue, scientific = TRUE)

fit <- survfit(Surv(futime, fustat) ~ risk, data = rt)

summary(fit)

pdf(file="survivalTrain.pdf",width=5.5,height=5)

plot(fit,

lwd=2,

col=c("red","blue"),

xlab="Time (year)",

ylab="Survival rate",

main=paste("Survival curve (p=", pValue ,")",sep=""),

mark.time=T)

legend("topright",

c("high risk", "low risk"),

lwd=2,

col=c("red","blue"))

dev.off()

rt=read.table("riskTest.txt",header=T,sep="\t")

diff=survdiff(Surv(futime, fustat) ~risk,data = rt)

pValue=1-pchisq(diff$chisq,df=1)

pValue=signif(pValue,4)

pValue=format(pValue, scientific = TRUE)

fit <- survfit(Surv(futime, fustat) ~ risk, data = rt)

summary(fit)

pdf(file="survivalTest.pdf",width=5.5,height=5)

plot(fit,

lwd=2,

col=c("red","blue"),

xlab="Time (year)",

ylab="Survival rate",

main=paste("Survival curve (p=", pValue ,")",sep=""),

mark.time=T)

legend("topright",

c("high risk", "low risk"),

lwd=2,

col=c("red","blue"))

dev.off()

install.packages("survivalROC",dependencies = T)

library(survivalROC)

rt=read.table("riskTrain.txt",header=T,sep="\t",check.names=F,row.names=1)

pdf(file="rocTrain.pdf",width=6,height=6)

par(oma=c(0.5,1,0,1),font.lab=1.5,font.axis=1.5)

roc=survivalROC(Stime=rt$futime, status=rt$fustat, marker = rt$riskScore,

predict.time =1, method="KM")

plot(roc$FP, roc$TP, type="l", xlim=c(0,1), ylim=c(0,1),col='red',

xlab="False positive rate", ylab="True positive rate",

main=paste("ROC curve (", "AUC = ",round(roc$AUC,1),")"),

lwd = 2, cex.main=1.3, cex.lab=1.2, cex.axis=1.2, font=1.2)

abline(0,1)

dev.off()

roc=survivalROC(Stime=rt$futime, status=rt$fustat, marker = rt$riskScore,

predict.time =3, method="KM")

plot(roc$FP, roc$TP, type="l", xlim=c(0,1), ylim=c(0,1),col='green',

xlab="False positive rate", ylab="True positive rate",

main=paste("ROC curve (", "AUC = ",round(roc$AUC,3),")"),

lwd = 2, cex.main=1.3, cex.lab=1.2, cex.axis=1.2, font=1.2)

abline(0,1)

dev.off()

roc=survivalROC(Stime=rt$futime, status=rt$fustat, marker = rt$riskScore,

predict.time =5, method="KM")

plot(roc$FP, roc$TP, type="l", xlim=c(0,1), ylim=c(0,1),col='red',

xlab="False positive rate", ylab="True positive rate",

main=paste("ROC curve (", "AUC = ",round(roc$AUC,5),")"),

lwd = 2, cex.main=1.3, cex.lab=1.2, cex.axis=1.2, font=1.2)

abline(0,1)

dev.off()

rt=read.table("riskTest.txt",header=T,sep="\t",check.names=F,row.names=1)

pdf(file="rocTest.pdf",width=6,height=6)

par(oma=c(0.5,1,0,1),font.lab=1.5,font.axis=1.5)

roc=survivalROC(Stime=rt$futime, status=rt$fustat, marker = rt$riskScore,

predict.time =1, method="KM")

plot(roc$FP, roc$TP, type="l", xlim=c(0,1), ylim=c(0,1),col='red',

xlab="False positive rate", ylab="True positive rate",

main=paste("ROC curve (", "AUC = ",round(roc$AUC,3),")"),

lwd = 2, cex.main=1.3, cex.lab=1.2, cex.axis=1.2, font=1.2)

abline(0,1)

dev.off()

rt=read.table("riskTrain.txt",header=T,sep="\t",check.names=F,row.names=1)

rt=rt[order(rt$riskScore),]

riskClass=rt[,"risk"]

lowLength=length(riskClass[riskClass=="low"])

highLength=length(riskClass[riskClass=="high"])

line=rt[,"riskScore"]

line[line>10]=10

pdf(file="riskScoreTrain.pdf",width = 12,height = 5)

plot(line,

type="p",

pch=20,

xlab="Patients (increasing risk socre)",

ylab="Risk score",

col=c(rep("green",lowLength),

rep("red",highLength)))

trainMedianScore=median(rt$riskScore)

abline(h=trainMedianScore,v=lowLength,lty=2)

dev.off()

rt=read.table("riskTest.txt",header=T,sep="\t",check.names=F,row.names=1)

rt=rt[order(rt$riskScore),]

riskClass=rt[,"risk"]

lowLength=length(riskClass[riskClass=="low"])

highLength=length(riskClass[riskClass=="high"])

line=rt[,"riskScore"]

line[line>10]=10

pdf(file="riskScoreTest.pdf",width = 12,height = 5)

plot(line,

type="p",

pch=20,

xlab="Patients (increasing risk socre)",

ylab="Risk score",

col=c(rep("green",lowLength),

rep("red",highLength)))

abline(h=trainMedianScore,v=lowLength,lty=2)

dev.off()

rt=read.table("riskTrain.txt",header=T,sep="\t",check.names=F,row.names=1)

rt=rt[order(rt$riskScore),]

riskClass=rt[,"risk"]

lowLength=length(riskClass[riskClass=="low"])

highLength=length(riskClass[riskClass=="high"])

color=as.vector(rt$fustat)

color[color==1]="red"

color[color==0]="green"

pdf(file="survStatTrain.pdf",width = 12,height = 5)

plot(rt$futime,

pch=19,

xlab="Patients (increasing risk socre)",

ylab="Survival time (years)",

col=color)

abline(v=lowLength,lty=2)

dev.off()

rt=read.table("riskTest.txt",header=T,sep="\t",check.names=F,row.names=1)

rt=rt[order(rt$riskScore),]

riskClass=rt[,"risk"]

lowLength=length(riskClass[riskClass=="low"])

highLength=length(riskClass[riskClass=="high"])

color=as.vector(rt$fustat)

color[color==1]="red"

color[color==0]="green"

pdf(file="survStatTest.pdf",width = 12,height = 5)

plot(rt$futime,

pch=19,

xlab="Patients (increasing risk socre)",

ylab="Survival time (years)",

col=color)

abline(v=lowLength,lty=2)

dev.off()

**#risk curve**

install.packages("pheatmap",dependencies = T)

library(pheatmap)

rt=read.table("riskTrain.txt",sep="\t",header=T,row.names=1,check.names=F)

rt=rt[order(rt$riskScore),]

rt1=rt[c(3:(ncol(rt)-2))]

rt1=t(rt1)

rt1=log2(rt1+0.01)

annotation=data.frame(type=rt[,ncol(rt)])

rownames(annotation)=rownames(rt)

pdf(file="heatmapTrain.pdf",width = 12,height = 5)

pheatmap(rt1,

annotation=annotation,

cluster_cols = FALSE,

fontsize_row=11,

fontsize_col=3,

color = colorRampPalette(c("green", "black", "red"))(50) )

dev.off()

rt=read.table("riskTest.txt",sep="\t",header=T,row.names=1,check.names=F)

rt=rt[order(rt$riskScore),]

rt1=rt[c(3:(ncol(rt)-2))]

rt1=t(rt1)

rt1=log2(rt1+0.01)

annotation=data.frame(type=rt[,ncol(rt)])

rownames(annotation)=rownames(rt)

pdf(file="heatmapTest.pdf",width = 12,height = 5)

pheatmap(rt1,

annotation=annotation,

cluster_cols = FALSE,

fontsize_row=11,

fontsize_col=3,

color = colorRampPalette(c("green", "black", "red"))(50) )

dev.off()

**#** **Independent prognostic factors and nomogram**

setwd("C:\\Users\\BOSS\\Desktop\\last\\loop\\NOMOGRAM")

library(survival)

rt=read.table("indepInput.txt",header=T,sep="\t",check.names=F,row.names=1)

rt$futime=rt$futime/12

outTab=data.frame()

for(i in colnames(rt[,3:ncol(rt)])){

cox <- coxph(Surv(futime, fustat) ~ rt[,i], data = rt)

coxSummary = summary(cox)

coxP=coxSummary$coefficients[,"Pr(>|z|)"]

outTab=rbind(outTab,

cbind(id=i,

HR=coxSummary$conf.int[,"exp(coef)"],

HR.95L=coxSummary$conf.int[,"lower .95"],

HR.95H=coxSummary$conf.int[,"upper .95"],

pvalue=coxSummary$coefficients[,"Pr(>|z|)"])

)

}

write.table(outTab,file="uniCox.xls",sep="\t",row.names=F,quote=F)

install.packages("Hmisc",dependencies = T)

install.packages("lattice",dependencies = T)

install.packages("Formula",dependencies = T)

install.packages("ggplot2",dependencies = T)

install.packages("foreign",dependencies = T)

install.packages("rms",dependencies = T)

library(rms)

rt=read.table("risk.txt",sep="\t",header=T,row.names=1,check.names=F)

rt=rt[c(1:(ncol(rt)-2))]

dd <- datadist(rt)

options(datadist="dd")

f <- cph(Surv(futime, fustat) ~ ., x=T, y=T, surv=T, data=rt, time.inc=1)

surv <- Survival(f)

nom <- nomogram(f, fun=list(function(x) surv(1, x), function(x) surv(3, x), function(x) surv(5, x)),

lp=F, funlabel=c("3-year survival", "5-year survival"),

maxscale=100,

fun.at=c(0.99, 0.9, 0.8, 0.7, 0.6, 0.5, 0.4, 0.3,0.2,0.1,0.05))

pdf(file="nomogram.pdf",height=6,width=10)

plot(nom)

dev.off()

install.packages("survminer",dependencies = T)

library(survival)

library(survminer)

rt=read.table("indepInput.txt",header=T,sep="\t",check.names=F,row.names=1)

rt$futime=rt$futime/12

multiCox=coxph(Surv(futime, fustat) ~ ., data = rt)

multiCox=step(multiCox,direction = "both")

multiCoxSum=summary(multiCox)

outTab=data.frame()

outTab=cbind(

HR=multiCoxSum$conf.int[,"exp(coef)"],

HR.95L=multiCoxSum$conf.int[,"lower .95"],

HR.95H=multiCoxSum$conf.int[,"upper .95"],

pvalue=multiCoxSum$coefficients[,"Pr(>|z|)"])

outTab=cbind(id=row.names(outTab),outTab)

write.table(outTab,file="multiCox.xls",sep="\t",row.names=F,quote=F)

pdf(file="forest.pdf",

width = 8,

height = 5,

)

ggforest(multiCox,

main = "Hazard ratio",

cpositions = c(0.02,0.22, 0.4),

fontsize = 0.7,

refLabel = "reference",

noDigits = 2)

dev.off()

**# calibration**

setwd("C:\\Users\\BOSS\\Desktop\\check\\new nomogram\\final test\\calibration")

library(rms)

library(foreign)

library(survival)

seer<-read.table("indepInput.txt",header=T,sep="\t",check.names=F,row.names=1)

cox3 <- cph(Surv(futime,fustat) ~ stage +riskScore ,surv=T,x=T, y=T,time.inc = 1*12*3,data=seer)

cal <- calibrate(cox1, cmethod="KM", method="boot", u=1*12*3, m=48, B=100)

pdf("calibrate3.pdf",12,8)

par(mar = c(10,5,3,2),cex = 1.0)

plot(cal,lwd=3,lty=2,errbar.col="black",xlim = c(0,1),ylim = c(0,1),xlab ="Nomogram-Predicted Probability of 1-Year Survival",ylab="Actual 1-Year Survival",col="blue")

lines(cal,c('mean.predicted','KM'),type = 'a',lwd = 3,col ="black" ,pch = 16)

box(lwd = 1)

abline(0,1,lty = 3,lwd = 3,col = "black")

dev.off()

cox5 <- cph(Surv(futime,fustat) ~ stage +riskScore ,surv=T,x=T, y=T,time.inc = 1*12*5,data=seer)

cal <- calibrate(cox5, cmethod="KM", method="boot", u=1*12*5, m=48, B=100)

pdf("calibrate3.pdf",12,8)

par(mar = c(10,5,3,2),cex = 1.0)

plot(cal,lwd=3,lty=2,errbar.col="black",xlim = c(0,1),ylim = c(0,1),xlab ="Nomogram-Predicted Probability of 1-Year Survival",ylab="Actual 1-Year Survival",col="blue")

lines(cal,c('mean.predicted','KM'),type = 'a',lwd = 3,col ="black" ,pch = 16)

box(lwd = 1)

abline(0,1,lty = 3,lwd = 3,col = "black")

dev.off()

**#C-index**

setwd("C:\\Users\\BOSS\\Desktop\\check\\new nomogram\\final test\\C-index")

library(rms)

library(foreign)

library(survival)

seer<-read.table("riskTest.txt",header=T,sep="\t",row.names = 1)

ddist <- datadist(seer)

options(datadist='ddist')

cox2=coxph(Surv(futime, fustat) ~ ., data = seer)

coxpe <- predict(cox2)

c_index=1-rcorr.cens(coxpe,Surv(seer$futime,seer$fustat))

c_index
